# The Temporal Dynamics of Attention to Threat and GAD Symptoms: A Study of LPP Slopes

**DOI:** 10.1111/psyp.70157

**Published:** 2025-09-30

**Authors:** Ben Swanson, Matt R. Judah, Grant S. Shields

**Affiliations:** ^1^ Department of Psychological Science University of Arkansas Fayetteville Arkansas USA

**Keywords:** generalized anxiety disorder, late positive potential, LPP slopes

## Abstract

Individuals experiencing generalized anxiety disorder (GAD) show heightened attention to threat, as suggested by greater amplitude of the late positive potential (LPP). However, amplitude measurements do not fully capitalize on the high temporal resolution of EEG. Specifically, amplitude does not reflect the rate of change in the LPP over a window of interest, which may be important to understand LPP dynamics in individuals with GAD. Indeed, this rate of change of the LPP (i.e., LPP slope) may reflect attentional orienting. The current study leveraged multilevel models to examine the LPP in relation to GAD symptoms. We hypothesized that more‐positive LPP slopes to threat images will be associated with GAD symptoms from 400 to 700 ms. Participants (*N* = 105) passively viewed blocks consisting of threatening or neutral images during EEG recording. Participant‐level LPP slopes were estimated using and extracted from multilevel models, and the extracted slopes were examined. LPP slopes were reliable, but they only weakly correlated with mean amplitudes—suggesting LPP slopes may capture an attentional process that could be distinct from that captured by mean amplitudes. When considered as concurrent predictors of GAD, in an early window of the LPP (400–700 ms), the conjunction of the threat‐LPP slope and the threat‐LPP mean amplitude explained three times as much variance in GAD symptoms as mean amplitude did alone. During a later window of the LPP (700–2000 ms), more‐negative LPP slope responses to threat were also related to GAD symptomology. Sensitivity analyses demonstrated that the relationship between the threat‐LPP slope and GAD symptoms was largely robust to measurement confounds. Together, the current study is the first to identify that LPP slope is uniquely related to GAD symptoms. Our data further suggest that LPP slope is a unique measure of the broader LPP response that warrants further investigation.

## Impact Statement

This is the first study to show that threat‐evoked rates of change of the late positive potential (i.e., LPP slopes) are uniquely associated with generalized anxiety disorder (GAD) symptoms. LPP slopes extracted from multilevel models explained three times more variance in GAD symptoms than mean amplitude alone. We demonstrate LPP slopes as an important new indicator of GAD vulnerability that warrants further investigation.

## 1 Introduction

Heightened attention to threat has been consistently associated with generalized anxiety disorder (GAD), a condition characterized by chronic, excessive, and uncontrollable worry (APA 2022). Behaviorally, GAD symptoms have been linked to faster reaction times to threatening stimuli (Goodwin et al. 2017). For individuals with GAD symptoms, threatening images also evoke greater amplitude in the late positive potential (LPP), an event‐related potential (ERP) associated with the motivational and emotional relevance of stimuli (Hajcak and Foti 2020). A large literature linking the LPP to GAD has grown over the last decade, clarifying the role of attention in the affective processing mechanisms that underlie GAD (Mennin et al. 2002, 2005), such as emotional reactivity and regulation. However, current approaches, typically based on averaging the LPP response across periods of 300 milliseconds or more, may be limited in capturing the fast dynamics of attention. We addressed this gap with a relatively novel analytic approach: examining LPP slopes to understand how rapid changes in LPP modulation relate to GAD symptoms.

The LPP is a centro‐parietal ERP that begins around 300 ms after visual stimulus onset, and it is often elicited via passive viewing paradigms (Cuthbert et al. 2000; Dunning and Hajack 2009; Hamrick et al. 2024; Schupp et al. 2024; Schupp and Kirmse 2021). LPP amplitude is thought to index attention to motivationally significant stimuli and is often larger for more emotionally salient stimuli (e.g., mutilation images) (Cuthbert et al. 2000; Hajcak and Foti 2020; Schupp et al. 2024; Schupp and Kirmse 2021). In support of this emotional‐attention interpretation, LPP amplitude increases when participants fixate on emotionally evocative regions of images and reduces when they fixate on less emotionally evocative regions (Dunning and Hajack 2009). Further supporting the emotional‐attention interpretation, and crucial for understanding the LPP in relation to GAD, various emotion regulation strategies (e.g., reappraisal) can alter or attenuate LPP amplitude to negatively valenced images (Hajcak and Nieuwenhuis 2006; Thiruchselvam et al. 2011). Despite some complexity in the literature (for a review, see MacNamara et al. 2022), the findings described above have led most to agree that LPP amplitude is a neural marker of attention to emotionally salient stimuli.

As might be expected from the idea that LPP amplitude indexes the modulation of attention by emotional processes, numerous studies have found that GAD is related to heightened LPP amplitude in response to threat stimuli in both clinical and non‐clinical samples assessing GAD symptoms (Judah et al. 2025; MacNamara et al. 2016; MacNamara and Hajcak 2009, 2010; MacNamara and Proudfit 2014). Notably, however, there are inconsistencies within the literature (e.g., Botelho et al. 2023; Weinberg and Hajcak 2011). Such inconsistencies may stem from an incomplete assessment of the LPP. In particular, most studies examining LPP‐GAD associations use the mean amplitude of the LPP, often by isolating that amplitude through difference waves (i.e., subtracting mean amplitude on neutral trials from mean amplitude on threat trials) or residual‐based scores (i.e., the residuals of a regression in which mean amplitude on neutral trials predicts mean amplitude on threat trials) (MacNamara et al. 2016; MacNamara and Hajcak 2010; Weinberg and Hajcak 2011). Within these approaches, the mean amplitude is thought to track the extent to which threatening stimuli convey greater attentional importance than neutral stimuli. However, mean amplitude approaches do not directly examine how LPP amplitude changes over the course of the measurement window (e.g., orienting, regulation, etc.), which is often 300 milliseconds or more. If the relation between mean amplitude and GAD is contingent upon the duration of the LPP increase, or its rate of increase, then examining mean amplitude in isolation may produce inconsistent results and fail to uncover dynamic changes in attention evoked by threat.

Given that the mean amplitude is insensitive to change across a measurement window of the LPP, there is a need for new approaches to measure the rate of change in LPP amplitude. If the LPP reflects attention, as is thought, then reliable measures of change could be reasonably assumed to reflect changes in attention. We refer to this change in amplitude over time as the LPP slope. The LPP slope is conceptually distinct from traditional measurement approaches of the LPP. As discussed, the LPP slope does not represent mean amplitude, but rather the change in amplitude over a period of interest. Further, where LPP latency captures the moment at which a particular ERP feature (e.g., the peak in the case of peak latency) is present at a particular window, the slope reflects the overall rate of change in amplitude. Together, the LPP slope may reflect the rate at which attention is garnered to a salient stimulus, making it conceptually distinct from both mean amplitude and latency measurement.

There are at least three reasons to expect that rates of change in the LPP (i.e., LPP slopes) might relate to anxiety. First, rapid engagement of emotional attention is theoretically important to anxiety. For example, someone who more rapidly engages attention to threat may be more likely to have a faster and more intense emotional response (Gross 2015). Consistent with the emotion dysregulation model of anxiety, a more intense emotional response would serve as a regulatory challenge, which may lead an individual with anxiety to turn to maladaptive strategies, such as worry or avoidance, to cope with intense emotion (Mennin et al. 2005). Second, distinct forms of emotion regulation (e.g., distraction and reappraisal) appear to differentially influence the LPP across time (Thiruchselvam et al. 2011), entailing that anxiety—which is linked to differences in emotion regulation strategies (Mennin et al. 2005)—might be associated with a different rate of LPP response. Third, emotion regulation strategies, such as cognitive reappraisal, have been shown to alleviate anxiety symptoms (Smits et al. 2012), and those who show a strong initial LPP response to a stimulus more readily choose to distract themselves from said stimulus rather than engage in a more cognitively demanding emotion regulation strategy (e.g., reappraisal) (Adamczyk et al. 2024). Thus, a better understanding of the time course of the LPP response—perhaps most particularly, its initial rate of change—might complement mean amplitude in indexing emotional attention and thus delineate a clearer neural basis of GAD.

To our knowledge, only a small number of studies have examined slopes of ERP components. Ghaderi‐Kangavari et al. (2023) used the slope of the centroparietal positivity (CPP), conceptually similar to the LPP, as a measure of evidence accumulation during a perceptual decision‐making task. More relevant to the current study, Monopoli et al. (2022) used a mixed modeling approach to investigate the LPP in relation to self‐reported emotion regulation (Monopoli et al. 2022). The authors found that individuals who reported more intense emotions had higher initial emotion reactivity (i.e., the LPP intercept measured at 500 ms) and reduced LPP slope from 500 ms to 2000 ms post‐stimuli presentation. Their results suggest that those who have a more intense emotional response tend to later avoid stimuli as measured by the LPP slope (Monopoli et al. 2022). Notably, the authors did not report examining the LPP slope in a window more aligned with the initial deployment of attention. Further, they did not examine LPP slopes in relation to a measure of anxiety. Nonetheless, these studies illustrate the potential of examining LPP slopes as an auxiliary of mean amplitude.

The literature described above highlights the potential importance and utility of examining LPP slopes in relation to GAD symptoms. To date, however, no study to our knowledge has examined this. To address this gap, the current study recorded EEG while participants passively viewed either neutral or threatening images. We then tested whether participant‐level changes in the LPP (i.e., LPP slopes) were associated with GAD symptoms. Specifically, we used multilevel models to extract participant‐level best linear unbiased estimates (i.e., LPP slopes) as measures of LPP change to both threatening and neutral images (Volpert‐Esmond et al. 2021). This approach allows participant‐level LPP slopes to be extracted and examined alongside traditional LPP measures (i.e., mean amplitude) in relation to GAD symptoms. To account for measurement confounds, we also assessed several potential alternative explanations for effects involving the slopes, including (a) LPP latency and (b) early activity preceding the LPP that may have altered the slope. Drawing on the literature described above, we predicted that individuals who report greater GAD symptoms would also show more‐positive threat‐LPP slopes during an early window (400–700 ms) of LPP modulation, aligning with the deployment of attention. We also examined the LPP slopes across a later window (700–2000 ms), predicting that GAD symptoms would be associated with a more‐negative threat‐LPP slope in this window.

## 2 Method

### 2.1 Participants

Participants were undergraduate students recruited from a psychology research participant pool at a large southeastern university between 2017 and 2020. The final sample with measurable EEG consisted of 105 participants (*M*_years_ = 20.48, SD = 3.74, 73.3% female). Participants had the following racial composition: Black (17%), White (70%), East Asian (10%), Latine (18%), South Asian (4%), Native American (3%), and Pacific Islander (1%), and were allowed to select multiple racial and ethnic backgrounds that best describe them. Participants spoke English and were at least 18 years old. These participants are further described elsewhere (Judah et al. 2025). Further, the current study was co‐registered (https://aspredicted.org/cqb7‐kbs9.pdf).

### 2.2 Materials

#### 2.2.1 Anxiety Symptoms

The seven‐item Generalized Anxiety Disorder‐7 (GAD‐7) was used to assess anxiety symptoms (Spitzer et al. 2006). Participants were asked how often they were bothered by the items (e.g., “Feeling nervous, anxious, or on edge”) over the last two weeks. Participants responded on a 4‐point Likert scale from 0 (not at all) to 3 (nearly every day). Scores across all items were summed to create a total score for anxiety that was used in subsequent analyses. The GAD‐7 demonstrated good internal consistency, *α* = 0.86, and 32.6% of the sample scored at or above the clinical cutoff of 10. Participants were included in analyses regardless of whether they scored above the clinical cutoff. Of the 105 participants with complete EEG, 86 participants also had data for GAD symptoms.^1^ As such, models that examined GAD symptoms were fit using all 86 participants with GAD‐7 data.

### 2.3 Procedure

All procedures for the study were approved by the institutional review board. Participants first provided informed consent and proceeded to complete several questionnaires, including the GAD‐7. Participants were then fitted with a custom 33‐channel EEG cap manufactured by BioSemi. Additional electrodes were placed below the left eye, at the outer canthus of each eye, and at each mastoid. Stimuli were presented on a monitor (Dell S2716DG, 27.0‐in. 60 Hz, 1024 × 768 resolution) that was positioned at 70 cm from the eye of the participant with Presentation software (v. 19.0; Neurobehavioral Systems Inc., Berkley, CA, www.neurolabs.com). Participants completed a passive viewing task using images from the International Affective Picture System^2^ (Lang et al. 2008). Participants viewed stimuli in 6 randomized blocks, each containing 20 images that were either neutrally or negatively valenced (60 negative, 60 neutral, 120 total). The images within each block were all neutrally or negatively valenced and randomized. For each image, participants were instructed to fixate on a fixation cross lasting 500 ms that was followed by an image presented for 2000 ms. Intertrial intervals varied randomly from three options of 800, 1000, and 1200 ms. The images were presented on a gray background (RGB: 192, 192, 192) with a visual angle of 25.36 × 19.06.

### 2.4 EEG Preprocessing

EEG was recorded at a sampling rate of 1024 Hz using a BioSemi ActiveTwo system. Data were subsequently processed offline using both EEGLAB and ERPLAB (Delorme and Makeig 2004; Lopez‐Calderon and Luck 2014). Data were referenced to the average of both mastoids and filtered with a 0.1 Hz high‐pass filter. Independent component analysis (ICA) was conducted to identify ocular artifacts, which were then manually removed. Data were then segmented from −200 to 2000 ms post stimulus onset and baselined referenced from −200 to 0 ms. Trials containing a blink within 200 ms of stimulus onset or containing values exceeding ±200 μV relative to the baseline period were excluded (Hamrick et al. 2024; Judah et al. 2025; Pegg et al. 2024). On average, 52.5 (SD = 4.9) trials were retained for neutral images and 51.4 (SD = 5.6) trials were retained for threat images.

### 2.5 Data Analysis

**FIGURE 1:**
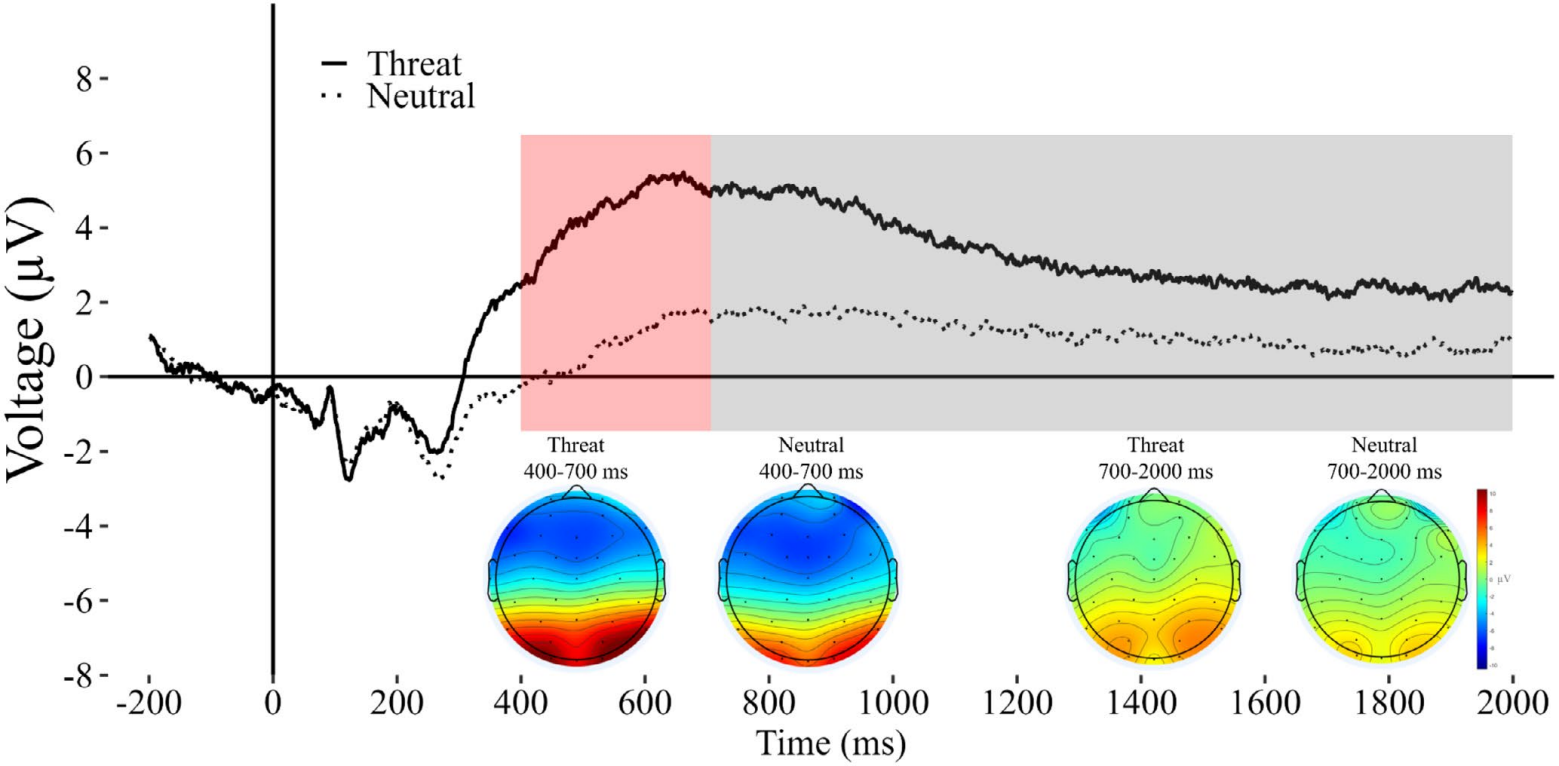
Grand average waveform with scalp maps. Grand average waveform measured at Pz. Scalp topographies for threatening and neutral images for each measurement window are also shown.

The ERP waveform for each participant was created by averaging trials across each image valence (neutral and threat) at Pz, consistent with prior work (Judah et al. 2025; McGhie et al. 2021; Weinberg et al. 2021). The measurement at Pz is also consistent with prior work that has shown the maximum effect of emotional valence is at Pz (Liu et al. 2012). Specifically, for each participant, we identified the average amplitude at each time point in the LPP response (e.g., 401 ms, 402 ms, etc.) across both neutral and threat trials. Across the entire window of the LPP (−200 to 2000 ms), for each participant, 2458 values for amplitude were extracted for both threatening and neutral images. The grand average wave is shown in Figure 1.

LPP amplitudes were analyzed with multilevel modeling using the nlme package (version 3.1‐162) in R version 4.3.1 (R Core Team 2023, Pinheiro and Bates 2023). Data were structured in long format, such that each participant had repeated observations for amplitude across time for each image valence (threat and neutral). Our primary variable of interest was each participant's rate of change (i.e., LPP slope) of the LPP over the time window of interest. We examined the early LPP from 400–700 ms, consistent with prior work (Grant et al. 2015, 2023; Hamrick et al. 2024; Moser et al. 2009). We deviated slightly from the co‐registration to additionally examine the later LPP window from 700–2000 ms to best capture the descending slope of the LPP throughout the duration of image presentation. The first value for time in each window was set at 0 (i.e., 400 ms was set to 0 for the 400–700 ms interval) to represent the intercept (Brush et al. 2018). A stepwise process for creating multilevel models is described in the Supporting Information. The best‐fitting model was fitted using an uncorrelated random effect structure with a restricted maximum likelihood estimator. The final model included fixed and random effects of the interaction between time (each sample post stimulus onset) and image valence (threat versus neutral). The final model is denoted below.

$$\begin{array}{c} \mathrm{LPP}\;\text{Amplitude}∼\text{Time}+\text{Image Valence}+\text{Time}\times \text{Image Valence}\\ +\left(1+\text{Image Valence}+\text{Time}+\text{Time}\times \text{Image Valence}\;|\;\text{Participant}\right) \end{array}$$

The best linear unbiased predictions (BLUPs) from the random effects were extracted to estimate the LPP slope for each image valence across each participant (Volpert‐Esmond et al. 2021). For clarity, we refer to the BLUPs as the LPP slope. The extracted slopes reflect the rate at which the LPP changed during a given time window of interest, for a given participant, for a given image valence. In addition to using the slopes, mean amplitudes were also calculated for both threat and neutral stimuli. The extracted slopes for each image valence, along with mean amplitudes, were added to stepwise linear regression models as predictors of anxiety symptoms. Influential observations were identified using DFBETAs with a cutoff of 2÷√*N*.^3^

To estimate the reliability of the slope estimates, a linear model with time as a predictor of amplitude was estimated. From the linear model, the extracted coefficient for time represented the LPP slope for a given trial. Through estimating a slope for each trial, it was possible to follow the approach from Luck et al. (2021) and calculate the $\hat{\mathrm{SME}}$ for each participant. To estimate group‐level reliability across image valences, the mean square of the $\hat{\mathrm{SME}}$ for both the threat and neutral conditions was calculated. To estimate overall reliability for each condition, we estimated reliability using the formula $\frac{{\hat{\mathrm{VAR}}}_{\text{Total}}-\mathrm{MS}\left(\hat{\mathrm{SME}}\right)}{{\hat{\mathrm{VAR}}}_{\text{Total}}}$, where ${\hat{\mathrm{VAR}}}_{\text{Total}}$ represents the squared standard deviation of all scores (Luck et al. 2021). From 400–700 ms, reliability of the slope estimate was good for both the threat 0.84 and neutral conditions 0.86. From 700–2000 ms, reliability was also good for both threat 0.85 and neutral 0.86 trials.

Several sensitivity analyses were also conducted to determine whether any relationship between the slope and anxiety was due to measurement confounds. First, to control for latency effects that may impact the slope measurement, the peak latency was calculated as the time point at which the amplitude measurement was at its maximum for both the early and late windows of the LPP. Second, we controlled for the influence of the Early Posterior Negativity (EPN) by calculating the average activity from the pool of the O1, O2, P7, and P8 electrodes from 150–300 ms (Farkas et al. 2020). Third, we also examined the amplitude value directly preceding the measurement window to determine whether the amplitude value at the starting point of the slope impacted the slope measurement. For example, a greater negative deflection in the waveform preceding the LPP could have also inflated slope values. As such, analyses were conducted to examine how far the amplitude measurement directly preceding the measurement window (i.e., 399 ms for the 400–700 ms window) differed from the mean amplitude during the rest of image viewing (i.e., the mean amplitude from 0–2000 ms). We refer to this early activity as the pre‐slope voltage below.

## 3 Results

### 3.1 Descriptives and Preliminary Analyses

Means, standard deviations, and bivariate correlations among both variables of interest and demographics are shown in Table 1. Consistent with expectations, we found that gender was associated with anxiety symptoms *β* = 0.36, *t*(84) = 3.49, *p* < 0.001, such that those who reported their gender as female reported more anxiety (*M* = 8.26, SD = 4.82) than those who identified as male (*M* = 4.05, SD = 4.39).

**TABLE 1:** Means, standard deviations, and correlations between study variables.

Variable	1	2	3	4	5	6	7	8	9	10	11	12	13
1. Threat intercept early	—												
2. Threat slope early	−0.58***	—											
3. Threat mean amplitude early	0.93***	−0.25*	—										
4. Neutral intercept early	0.78***	−0.48***	0.72***	—									
5. Neutral slope early	−0.41***	0.68***	−0.19†	−0.58***	—								
6. Neutral mean amplitude early	0.75***	−0.26*	0.78***	0.93**	−0.25*	—							
7. Threat intercept late	0.61***	0.15	0.79***	0.47***	0.07	0.59***	—						
8. Threat slope late	−0.26*	−0.18†	−0.39***	−0.31***	−0.03	−0.39***	−0.58***	—					
9. Threat mean amplitude late	0.60***	0.09	0.76***	0.40***	0.07	0.51***	0.91***	−0.19†	—				
10. Neutral intercept late	0.51***	0.02	0.62***	0.67***	0.05	0.83***	0.62***	−0.36***	0.56***	—			
11. Neutral slope late	−0.04	−0.10	−0.10	−0.18†	−0.08	−0.25*	−0.18†	0.32***	−0.05	−0.43***	—		
12. Neutral mean amplitude late	0.55***	−0.02	0.64***	0.66***	0.02	0.79***	0.60***	−0.23*	0.60***	0.89***	0.02	—	
13. GAD−7 Total	0.11	0.27*	0.25*	−0.01	0.20†	0.09	0.37***	−0.25*	0.31***	0.12	0.09	0.17	—
Mean	3.13	0.01	4.57	−0.47	0.01	0.74	4.77	< 0.01	3.20	1.72	< 0.01	1.22	7.2
SD	6.40	0.02	5.28	5.43	0.01	4.54	4.85	< 0.01	4.07	3.61	< 0.01	3.27	5.0

*Note:* “Early” refers to 400–700 ms, whereas “late” refers to 700–2000 ms. Gender is coded such that 0 = male and 1 = female.

*** *p* < 0.001.

** *p* < 0.01.

* *p* < 0.05.

^†^ *p* < 0.10 (two‐tailed).

### 3.2 Fixed Effects

#### 3.2.1 400–700 ms Window

From 400 to 700 ms, between‐subjects variability (i.e., individual differences) accounted for approximately 67% of the variance in LPP amplitude (*ICC* = 0.68). There was a fixed effect of time *t*(64362) = 5.60, *p* < 0.001, such that amplitudes became more positive over time from 400–700 ms. There was also a main effect of image valence, such that amplitudes were greater for threat (*M* = 4.45, SE = 0.53) than neutral images (*M* = 0.87, SE = 0.46), *t*(64362) = 8.45, *p* < 0.001. There was no significant interaction of Time × Image Valence, such that—surprisingly—the rate of change in amplitudes, on average and across all participants, did not differ between negative and neutral pictures during this time window, *t*(64362) = 1.13, *p* = 0.260 (see Table 2).

**TABLE 2:** Fixed effects of multilevel model.

	*b*	SE	df	*t*	*p*
*400–700 ms*					
(Intercept)	−0.29	0.55	64,362	−0.52	0.602
Time	0.01	< 0.01	64,362	5.60	< 0.001
Image valence	3.38	0.40	64,362	8.46	< 0.001
Time × Image valence	< 0.01	< 0.01	64,362	1.13	0.260
*700–2000 ms*					
(Intercept)	1.78	0.36	279,402	4.99	< 0.001
Time	< 0.01	< 0.01	279,402	−3.77	< 0.001
Image valence	2.99	0.38	279,402	7.92	< 0.001
Time × Image valence	< 0.01	< 0.01	279,402	−4.38	< 0.001

*Note:* Image valence was coded such that 0 = neutral and 1 = threat.

#### 3.2.2 700–2000 ms Window

From 700 to 2000 ms, between‐subjects variability (i.e., individual differences) accounted for approximately 57% of the variance in LPP amplitude (*ICC* = 0.57). There remained a fixed effect of time *t*(297402) = −3.77, *p* < 0.001, but in the opposite direction, such that amplitudes decreased over the time window. There remained a main effect of image valence, such that amplitudes were greater for threat (*M* = 3.22, SE = 0.39) than neutral images (*M* = 1.18, SE = 0.32), *t*(297402) = 9.33, *p* < 0.001. There was a significant Time × Image Valence interaction, such that LPP slopes during this later window were more negative for threat than neutral trials *t*(279402) = −4.38, *p* < 0.001—perhaps suggestive of recovery or emotion regulation.

### 3.3 Participant‐Level LPP Slope

#### 3.3.1 400–700 ms Window

**FIGURE 2:**
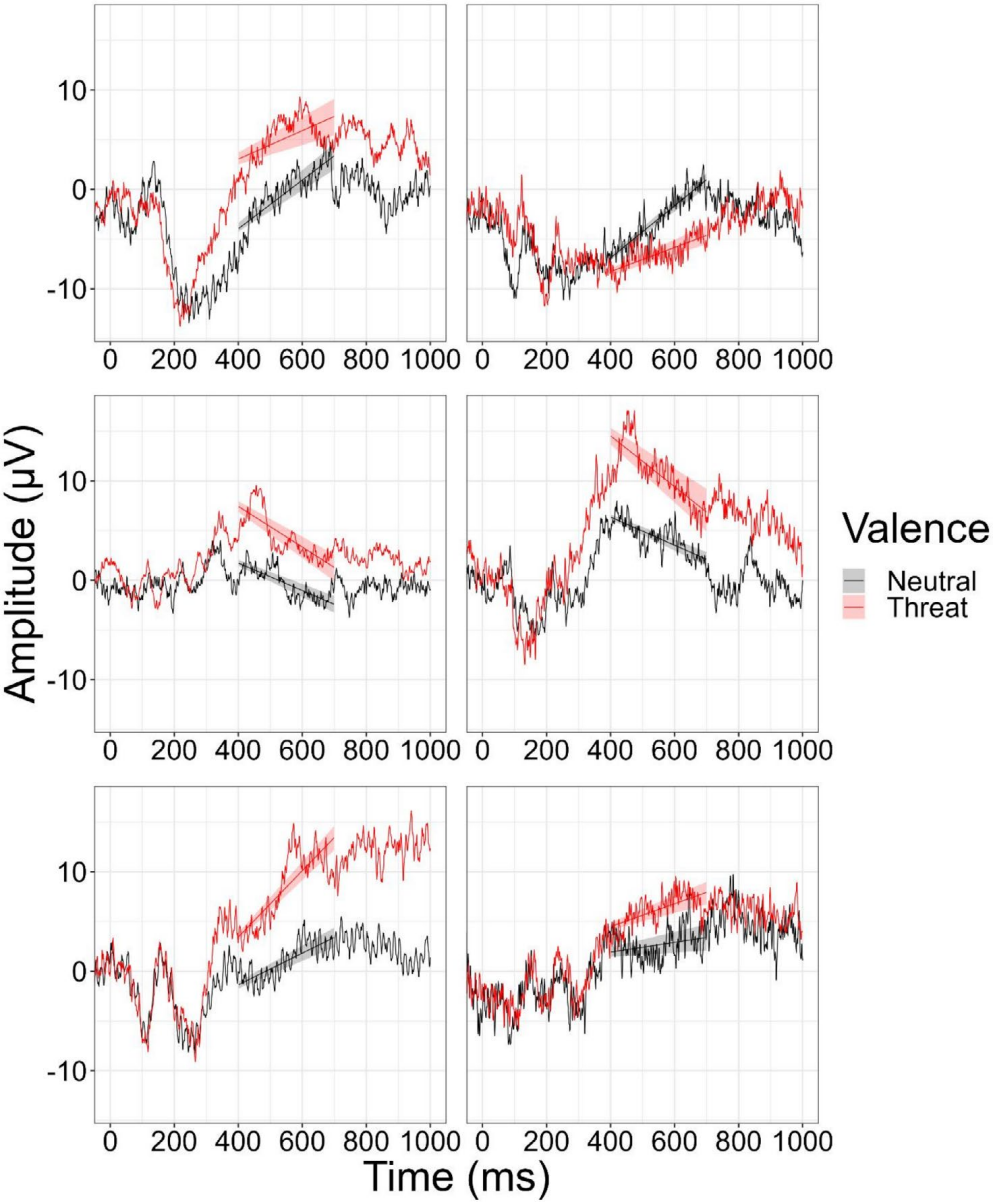
Participant‐level estimates for six participants. Extracted waveform for six of the 86 total participants, depicting the early window (400–700 ms) analysis of the LPP. The plotted lines are the estimated participant‐level effects from the multilevel model that were used as predictors of anxiety symptoms. A bootstrap analysis with 100 iterations was conducted to estimate the displayed 95% confidence intervals for the intercepts and slopes (shaded areas). For each iteration, 100 random amplitude values in the participant‐level averaged waveform were selected. The randomly selected values were sorted ascending by time. The data were then fit using the same multilevel model that included time and image valence as both fixed and random effects. For each iteration, the estimated participant‐level LPP slopes were extracted for both threat and neutral stimuli.

A subset of the extracted negative and neutral LPP slopes is depicted in Figure 2. Threat‐LPP slope was weakly correlated with threat mean amplitude (*r* = −0.25, *p* = 0.010). Neutral LPP slope was also weakly correlated with neutral mean amplitude (*r* = −0.25, *p* = 0.010) images.

Consistent with prior work on GAD and the LPP, threat mean amplitude was positively related to GAD symptoms, *β* = 0.25, *t*(84) = 2.39, *p* = 0.019. When further adding the threat‐LPP slope as a predictor to this model, both the threat‐LPP slope, *β* = 0.36, *t*(83) = 3.47, *p* < 0.001, and threat‐LPP mean amplitude, *β* = 0.35, *t*(83) = 3.37, *p* = 0.001, were positively related to GAD symptoms. Notably, the model that included both threat‐LPP slope and threat‐LPP mean amplitude explained significantly more variance than the model that included mean amplitude alone, Δ*R* ^2^ = 0.12, *p <* 0.001. Together, the model including both threat‐LPP slope and mean amplitude accounted for approximately 18% (*R* ^2^ = 0.18) of the variance in GAD symptoms. Subsequent models that added neutral slope and mean amplitude did not explain additional variance (Δ*R* ^2^ = 0.02), suggesting the effects were specific to threat images.^4^ Further, the association between threat‐LPP slope, *β* = 0.32, *t*(80) = 2.30, *p* = 0.024, and threat‐LPP mean amplitude, *β* = 0.43, *t*(80) = 2.59, *p* = 0.012, held after controlling for gender^5^ (*β* = 0.24, *t*(80) = 2.30, *p* = 0.024). Full results from the stepwise regression analyses are shown in Table 3.

**TABLE 3:** Stepwise regression results.

Predictor	400–700 ms				700–2000 ms			
	Model 1	Model 2	Model 3	Model 4	Model 1	Model 2	Model 3	Model 4
Threat mean amplitude	**0.25** *	**0.35** **	**0.53** **	**0.43** *	**0.31** **	**0.28** **	**0.33** *	**0.28** *
Threat Slope		**0.36** ***	**0.34** *	**0.32** *		−0.20†	**−0.24** *	**−0.22** *
Neutral mean amplitude			< 0.01	−0.18			−0.09	−0.08
Neutral Slope			−0.24	−0.04			0.15	0.17^†^
Gender				**0.24** *				**0.31** **
*R* ^2^	0.06	0.18	0.20	0.25	0.10	0.14	0.16	0.25
Δ*R* ^2^	0.04	**0.12** ***	0.02	**0.05** *	**0.10** **	0.04^†^	0.02	**0.09** **

*Note:* Standardized regression coefficients (*β*s) and *R* ^2^ are reported. *p* values < 0.05 are shown in bold. Gender was dummy coded such that male = 0 and female = 1.

*** *p* < 0.001.

** *p* < 0.01.

* *p* < 0.05.

^†^ *p* < 0.10 (two‐tailed).

#### 3.3.2 700–2000 ms Window

Bivariate correlations suggested that the threat‐LPP slope during the 700–2000 ms window was negatively associated with GAD symptoms, *r* = −0.25, *p* = 0.021, whereas threat‐LPP mean amplitude was positively related to GAD symptoms, *r* = 0.31, *p* = 0.003. In a linear model, threat‐LPP mean amplitude was related to GAD symptoms, *β* = 0.31, *t*(84) = 3.04, *p* = 0.003. Adding threat‐LPP slope to the model, threat‐LPP mean amplitude remained positively related, *β* = 0.28, *t*(83) = 2.71, *p* = 0.008, and threat slope was marginally negatively related to GAD symptoms, *β* = −0.20, *t*(83) = −1.92, *p* = 0.058.^6^ The threat‐LPP slope was negatively related to GAD symptoms even when neutral LPP slope and amplitude were included within the model, *β* = −0.24, *t*(81) = −2.25, *p* = 0.027.^7^ After including gender,^8^ neutral mean amplitude was also marginally related to GAD symptoms, *β* = −0.17, *t*(80) = 1.68, *p* = 0.094. The stepwise regression model for this later time window is shown in Table 3.

### 3.4 Sensitivity Analyses

#### 3.4.1 EPN

The EPN for threat images was calculated as the average activity from the pool of the O1, O2, P7, and P8 electrodes from 150–300 ms. The EPN was positively related to LPP mean amplitudes from 400–700 ms, *r =* 0.41, *p* < 0.001, and GAD symptoms, *r* = 0.22, *p* = 0.040; however, it was not related to LPP slopes from 400–700 ms, *r* = 0.02, *p* = 0.821. We then added both LPP slope to threat from 400–700 ms and EPN amplitude for threat images as predictors of GAD symptoms in a linear model. In this model, threat EPN mean amplitude and threat‐LPP slope were significant predictors of GAD symptoms (*β* = 0.22, *t*(83) = 2.12, *p =* 0.037, and *β* = 0.26, *t*(83) = 2.55, *p =* 0.013, respectively).

#### 3.4.2 Early Pz Amplitude

In the early LPP window, the pre‐slope voltage was associated with LPP slope to threat images during the 400–700 ms window, *r* = −0.53, *p* < 0.001. We then added both LPP slope to threat and early Pz amplitude for threat images as predictors of GAD symptoms in a linear model. In this model, both threat slope *β* = 0.56, *t*(83) = 3.66, *p* < 0.001 and pre‐slope voltage, *β* = −0.40, *t*(83) = −2.58, *p* = 0.021, were related to GAD symptoms. Finally, we followed the sensitivity analysis procedure described in the paragraph above for the 700–2000 ms time window. Within this window, neither slopes to threat images, *β* = −0.19, *t*(83) = −1.59, *p* = 0.120, nor early activity, *β* = −0.11, *t*(83) = −0.92, *p* = 0.361, were related to GAD symptoms.

#### 3.4.3 Latency

Within the 400–700 ms window, the threat‐LPP peak latency was positively correlated with the threat‐LPP slope, *r* = 0.68, *p* < 0.001. Specifically, later peak latencies were correlated with more‐positive threat‐LPP slopes. We then added both LPP slope for threat and LPP peak latency for threat images as predictors of GAD symptoms in a linear model. When both peak latency and the threat‐LPP slope were included as predictors of GAD symptoms, the LPP threat slope was related to GAD symptoms, *β* = 0.37, *t*(83) = 2.65, *p* = 0.010, whereas peak latency was not, *β* = −0.16, *t*(83) = −1.41, *p* = 0.257. These analyses demonstrate that while LPP slope and latency were related, the LPP slope explained unique variance in GAD symptoms, whereas latency did not.

It was possible that a combination of the amplitude preceding the measurement window, plus the peak latency, influenced the slope measurement. To determine whether this possibility may have influenced our results, we examined early Pz amplitude (the amplitude at 399 ms), the threat‐LPP slope, and peak latency as predictors of GAD symptoms. In this model, LPP slope to threat, *β* = 0.68, *t*(82) = 3.81, *p* < 0.001, and early Pz amplitude, *β* = −0.40, *t*(82) = −2.64, *p* = 0.009, were associated with GAD symptoms, whereas peak latency was not, *β* = −0.17, *t*(82) = −1.29, *p* = 0.201.

To further determine whether the slope may serve as a proxy for latency, we also estimated fractional area latency from 400–700 ms. Fractional area latency was assessed as the point at which the area under the positive curve reached 50%. LPP slope to threat images was weakly correlated with fractional area latency to threat, *r* = 0.28, *p* = 0.004. When both LPP slope to threat and fractional area latency to threat were assessed in relation to GAD symptoms, only LPP slope to threat was related to GAD symptoms, *β* = 0.25, *t*(83) = 2.36, *p* = 0.021—whereas fractional area latency was not, *β* = 0.04, *t*(83) = 0.36, *p* = 0.718.

## 4 Discussion

Heightened attention to threat has been consistently associated with GAD symptoms. However, no study to our knowledge has leveraged the temporal precision of ERPs to capture how fast‐changing attentional dynamics relate to GAD symptoms. To our knowledge, the current study is the first to show that rates of LPP change (i.e., LPP slopes) incrementally explain unique variance in GAD symptoms, above and beyond LPP mean amplitudes. Specifically, within an early LPP window (i.e., 400–700 ms), a more‐positive threat‐LPP slope was associated with greater GAD symptoms. Conversely, within a later LPP window (i.e., 700–2000 ms), a more‐negative LPP slope to threatening images was associated with greater GAD symptoms. In other words, threat‐LPP slope was uniquely associated with GAD symptoms. The threat‐LPP slope was an especially strong predictor of GAD symptoms in the early window of the LPP, where LPP slope and mean amplitude to threat together explained three times as much variance in GAD symptoms as assessing mean amplitude alone. Our results extend evidence that finds greater emotional reactivity in GAD (MacNamara et al. 2016; MacNamara and Hajcak 2010; MacNamara and Proudfit 2014) by highlighting the strength of association between GAD and a neural reactivity index per se rather than activity level alone.

At the neural level, it has been proposed that the LPP largely overlaps with the P300 and may reflect activity of the locus coeruleus (LC) norepinephrine system (NE) that relates to orienting (Hajcak and Foti 2020; Sara and Bouret 2012). As such, the LPP may reflect the degree to which the LC‐NE system is activated to orient attention (Hajcak and Foti 2020). Although this is a speculative interpretation of the neural basis of the LPP, a wealth of literature has shown that LPP amplitudes are larger for more attentionally significant stimuli (Cuthbert et al. 2000; for a review, see Hajcak and Foti 2020). Speculating further along these lines, our results could be taken to indicate that individuals higher in GAD symptoms show a greater rate of increase in attention to threatening stimuli within 400 to 700 ms after those stimuli appear.

The current study is one of the first to systematically examine the LPP slope. Importantly, we believe it is likely that the LPP slope captures an aspect of attention that is different from those captured by mean amplitude or latency. By definition, the LPP slope is the rate of change of LPP amplitude. Drawing upon the expansive LPP literature, if LPP amplitude reflects attention toward a salient stimulus, then the rate of change of amplitude may reflect the rate at which attention is modulated. Our data support this conceptual distinction as LPP slopes were only weakly correlated with mean amplitude, suggesting they may index separable aspects of attention. Further in support of this interpretation, LPP slope and mean amplitude explained unique variance in GAD symptoms.

**FIGURE 3:**
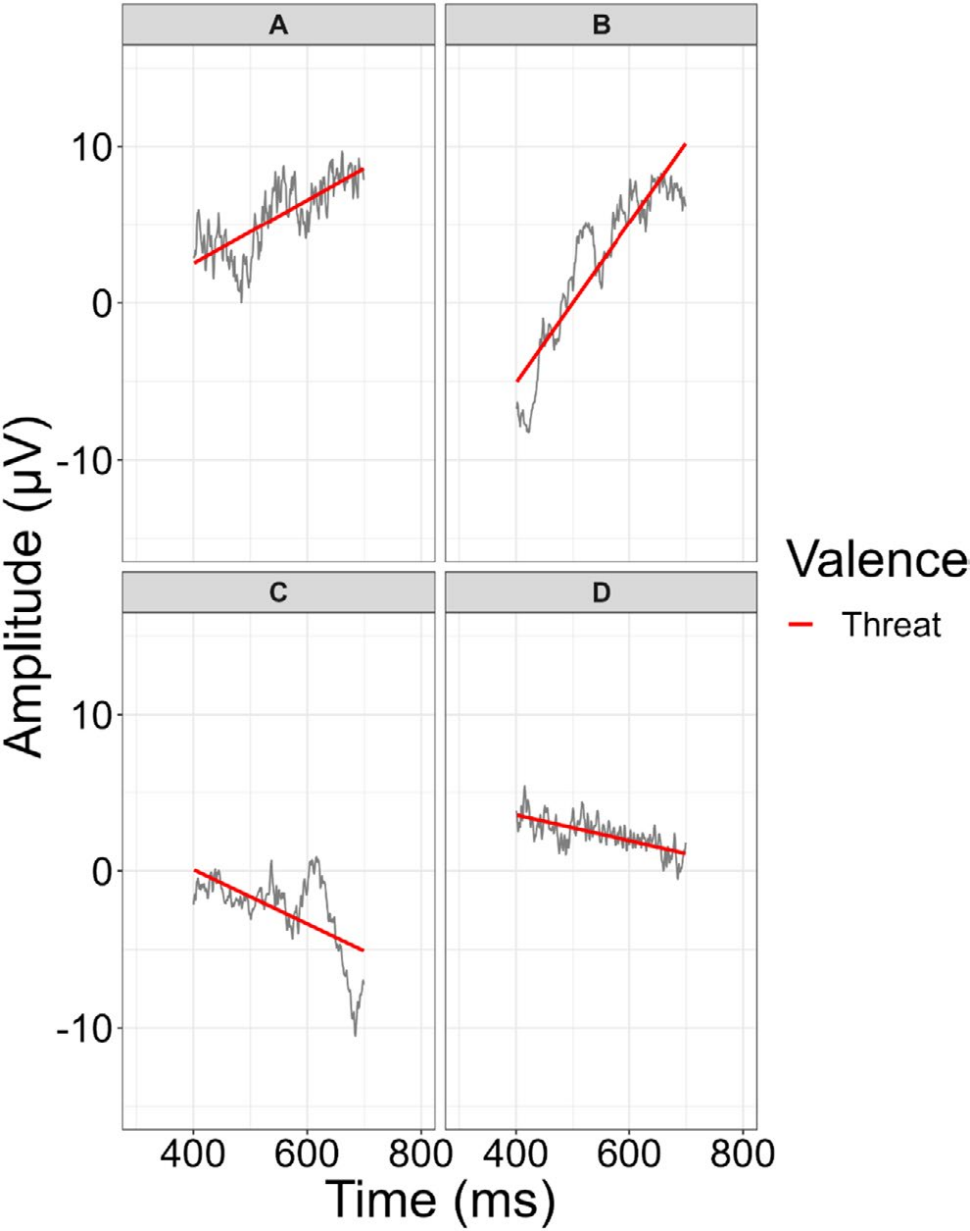
Visual distinction of LPP slope and LPP latency. The figure denotes four participants who were chosen to illustrate the association between the slope and latency. Within the 400–700 ms window, the threat‐LPP peak latency was positively correlated with the threat‐LPP slope, *r* = 0.68, *p* < 0.001. Those with earlier peak latencies (e.g., Participant D) tended to have lower (e.g., negative) slopes, whereas those with later peak latencies (e.g., Participants A and B) tended to have more‐positive slopes. Importantly, though, this was not always the case. For instance, Participant C had a peak latency just past 600 ms, and Participant D had a peak latency just past 400 ms, but their slopes were similar. Conversely, Participant A and Participant B both had peak latencies of 663 ms, and yet their LPP slopes differed. In our sensitivity analyses, we found that this difference is meaningful in relation to GAD symptoms. In particular, when both peak latency and the threat‐LPP slope were included as predictors of GAD symptoms, only the LPP slope predicted GAD symptoms.

Although the LPP slope is related to LPP latency, these measures are conceptually and statistically distinct. In particular, the LPP's latency indexes the point in time at which it occurs (e.g., the timing of the maximum amplitude in the case of peak latency), whereas the LPP slope indexes the change in amplitude over a window of interest. To highlight the distinction for further discussion, we illustrate some participants' LPP slopes and latencies in Figure 3, and we discuss them within the figure note.

Put simply, the latency of the LPP likely represents the point at which attention toward a stimulus in a window of interest is maximal. If the peak occurs early in a measurement window, the rate of change in amplitude is more likely to be negative, whereas if the peak of attention occurs later in a window, the rate of change in amplitude is more likely to be positive. However, LPP latency when assessed with fractional area was only weakly related to the LPP slope, which suggests they may capture different processes. Further, although both LPP slope and measures of LPP latency were correlated, only the threat slope was related to GAD symptoms. Ultimately, future research is needed to further understand the functional operation of the LPP slope. However, in our data, we demonstrate that the LPP slope and latency are conceptually and statistically distinct, and that the unique variance of the LPP slope appears to be important for understanding GAD symptoms.

Although more‐positive slopes to threat images were associated with GAD symptoms from 400–700 ms, more‐negative slopes to threat images were associated with GAD symptoms from 700–2000 ms. MacNamara recently proposed that, when confronted with a possible threatening stimulus, those on the anxiety spectrum are characterized by a heightened alarm system toward threat, which is then followed by disengagement from said threat (MacNamara 2025). Interestingly, our pattern of findings is remarkably consistent with her perspective (MacNamara 2025). The results in this latter LPP window also readily complement the only other study to our knowledge that has examined LPP slopes (Monopoli et al. 2022). In that study, Monopoli and colleagues examined LPP slopes in relation to self‐reported emotion dysregulation, in addition to other differences from the present study.^9^ Despite the methodological differences, combining the current results with the emotion regulation results obtained by Monopoli and colleagues paints a clear conceptual picture: heightened LPP responses to negative stimuli coupled with stronger LPP declines later predict maladaptive affective or anxiety‐related traits.

The similar pattern of LPP responses in our data and that of Monopoli et al. may suggest that LPP slopes in a passive viewing paradigm capture the time course of intrinsic emotion regulation (Monopoli et al. 2022). GAD is thought to be driven in part by maladaptive emotion regulation. Emotion regulation strategies are thought to require the engagement of executive control, or the ability to engage top‐down effort to resolve conflict. Executive control is a difficult process that requires effort, and this effort is inherently costly (Botvinick and Braver 2015; Shenhav et al. 2017). In line with this perspective, heightened attention to threat (i.e., as reflected by larger early LPP slopes and mean amplitudes) may require greater executive control to engage emotion regulation strategies. In the current study, threat‐LPP slope was associated with GAD symptoms at a timing that is synchronous to when emotion regulation strategies, such as reappraisal, begin to alter the LPP (i.e., 500–600 ms; Moser et al. 2009). Although the current study did not ask individuals to explicitly regulate their emotions, it is possible that the LPP slope and mean amplitudes from 400–700 ms reflect a point when individuals experiencing greater anxiety symptoms experience greater reactivity, which sets the stage for less effective emotion regulation.

The findings from the current study may complement past research to understand why, in part, individuals with GAD engage in maladaptive emotion regulation. Interestingly, individuals tend to choose cognitively easier strategies, such as distraction, when faced with high‐intensity stimuli that elicit larger LPP responses (Adamczyk et al. 2024; Dorman Ilan et al. 2019). Indeed, while a momentary distraction does effectively modify the LPP, distraction is less likely to modify LPP modulation to stimuli that are re‐encountered (Paul et al. 2016; Thiruchselvam et al. 2011). Considering the results from the current study, if individuals who report more GAD symptoms tend to perceive threatening stimuli with greater intensity (reflected by larger slopes and mean amplitudes), they may be less likely to engage in cognitively demanding strategies (i.e., reappraisal) that are more likely to habituate reactivity to threat. Specifically, individuals who report more GAD symptoms may be more likely to choose strategies (i.e., distraction) that maintain a heightened LPP response. Together, the heightened intensity through which individuals who report more GAD symptoms perceive threat may promote the choice of maladaptive strategies that eliminate the possibility for habituation and perpetuate maladaptive emotion regulation.

## 5 Strengths and Limitations

This study has a number of strengths, including extensive evaluation of potential confounds and alternative explanations. Importantly, the LPP slopes were reliable, with internal consistencies above 0.80. It was also possible that activity in the waveform prior to the measurement window could have been related to differences in slope measurement. To examine this possibility, sensitivity analyses were conducted that controlled for activity prior to the measurement window. We did find that, across both time windows, pre‐slope voltage was highly correlated with both LPP slope and mean amplitude. Importantly, though, in the early window of the LPP, the association between threat‐LPP slope and GAD symptoms was robust when controlling for activity preceding the measurement window from 400–700 ms. The association was only marginally significant in the later window (700–2000 ms), which suggests the association between GAD symptoms and slope may be more influenced by early LPP activity in this window. We also demonstrated through multiple sensitivity analyses that the LPP slope was related to but distinct from traditional LPP measurement techniques. The current study also recruited a moderate‐sized sample, with nearly a third of participants reporting GAD symptoms scoring above clinical cutoffs. Thus, the current study has the advantage of examining relatively novel analytic techniques on a moderately sized sample.

The current study is not without limitations. Although the sample size was relatively large, the participants were primarily White, undergraduate students, which limits conclusions to this population. Further, despite nearly a third of participants reporting GAD symptoms at or above clinical thresholds, we did not conduct diagnostic interviews. In the current study, participants are asked only to attend to the stimuli, meaning it is unclear how participants might be regulating their response. Regarding the slope measurement, the static measurement windows used in the current study may also limit interpretations. On average, the 400–700 ms window represented a period of an ascending slope. However, the LPP slope did not capture the ascension of the LPP for all participants, meaning the extracted slopes may capture different attentional processes across participants (e.g., increasing for some participants, decreasing for others). Although some have examined analytic methods that address individual‐level temporal and spatial differences (Schupp et al. 2024), no study to our knowledge has examined such an approach with LPP slope.

Despite the limitations, the current study opens numerous possibilities for future research. A pressing question for future work is to determine the attentional processes that LPP slopes represent. Other than the work of Monopoli & colleagues, few studies have investigated the slope of event‐related potentials in general. There has been some work examining the slope of a closely related component to the LPP, the Central Parietal Positivity (CPP) (Ghaderi‐Kangavari et al. 2023). For example, one study used CPP slopes in estimating evidence accumulation within a perceptual decision‐making task (Ghaderi‐Kangavari et al. 2023). In this framework, the increasing CPP response (i.e., slope) was thought to reflect increasing evidence accumulation toward a decision (Ghaderi‐Kangavari et al. 2023). While the current study can conclude that the threat‐LPP slope is related to anxiety, experimental designs are needed to hone the functional significance of the LPP slope. To address one limitation in the current study, future computational methods could determine the optimal time window to examine the LPP slope to fine‐tune when the ascension or descension of the LPP slope is maximal for a given individual. Regarding the measurement of GAD, further work investigating the facets of GAD is also warranted. GAD is a multi‐dimensional construct that consists of physiological (e.g., muscle tension), cognitive (e.g., worry), and emotional (e.g., irritability) symptoms (APA 2022). The measure used in the current study addresses overall anxiety symptoms. However, facets of anxiety (e.g., worry) could also be examined in relation to LPP slopes. The use of slope measurement need not be specific to GAD symptoms but may also be useful for understanding attentional processes related to other stimuli and individual differences (e.g., substance use cue reactivity).

## 6 Conclusion

The current study examined whether the rate of change of the LPP, as measured by the LPP slope, is related to GAD symptomology. We found that larger LPP slopes to threat images, especially during an early measurement window, explained a substantial amount of unique variance in anxiety symptoms, above and beyond mean amplitude measurement, even after controlling for possible measurement confounds. LPP slopes were weakly associated with mean amplitudes, which suggests that the LPP slope captures an aspect of the attentional response that is fundamentally different, but also critical, to GAD symptoms. Further, when controlling for LPP latency, only LPP slope to threat was related to GAD symptoms. In summary, the current study finds LPP slopes are a unique predictor of GAD symptoms and further proposes the LPP slope as an attentional process that warrants future investigation.

## Author Contributions

**Ben Swanson:** conceptualization, methodology, software, formal analysis, data curation, writing – original draft, visualization. **Matt R. Judah:** conceptualization, methodology, formal analysis, investigation, resources, writing – review and editing, supervision, project administration. **Grant S. Shields:** conceptualization, methodology, formal analysis, writing – review and editing, visualization.

## Conflicts of Interest

The authors declare no conflicts of interest.

## Supporting information


**Table S1:** Model fits for multilevel models.
**Table S2:** Stepwise regression results.
**Table S3:** Model results predicting GAD‐7 Sx across overlapping windows.

## Data Availability

The data that support the findings of this study are available from the corresponding author upon reasonable request.

## Endnotes

^1^
The current study was part of a larger study. The GAD‐7 was initially administered as part of the larger study, but the decision was made to remove it for later participants due to time constraints. As such, 19 participants were not administered the GAD‐7.

^2^
IAPS image numbers: Negative: 2688, 2730, 2800, 2900, 3030, 3060, 3100, 3101, 3120, 3170, 3180, 3181, 3191, 3220, 3230, 3261, 3266, 3350, 3500, 3530, 6212, 6213, 6260, 6350, 6360, 6510, 6560, 7380, 9040, 9050, 9250, 9252, 9253, 9300, 9321, 9340, 9400, 9410, 9419, 9420, 9428, 9435, 9500, 9520, 9530, 9560, 9570, 9571, 9600, 9800, 9810, 9900, 9901, 9903, 9910, 9921, 9922, 9925. Neutral: 2037, 2038, 2102, 2190, 2191, 2200, 2210, 2214, 2215, 2221, 2271, 2272, 2280, 2302, 2381, 2383, 2385, 2393, 2396, 2397,2440, 2441, 2480, 2493, 2495, 2499, 2512, 2514, 2516, 2570, 2595, 2749, 2840, 2850, 2870, 2880, 2890, 4000, 5390, 5410, 5471, 5510, 5726, 5731, 5740, 5800, 5870, 5875, 7002, 7004, 7025, 7100, 7130, 7234, 7290, 7493, 7550, 7590, 7700, 907.

^3^
In line with recommendations for use of DFBETAs (Aguinis et al. 2013), we adopted the standard cutoff for identifying influential observations via its DFBETA. In particular, when the DFBETA value was greater in absolute value than 2/sqrt(*N*), we considered it an outlier. In our case, our sample size *N* = 86, and thus 2/sqrt(*N*) = 2/sqrt(86) ~ = 2/9.27 = 0.2157. Therefore, whenever an observation's absolute DFBETA statistic was greater than 0.2157, we examined models that excluded that observation from analysis.

^4^
Removal of outliers from both threat mean amplitude and neutral LPP slope outliers revealed a negative association between neutral mean amplitude and GAD symptoms. No other relationships were affected by outliers.

^5^
The removal of threat LPP slope outliers led to a marginal negative association between neutral mean amplitude and GAD symptoms. No other relationships were affected by outliers.

^6^
The removal of outliers from threat LPP slope and mean amplitude strengthened the association between threat LPP slope and GAD symptoms.

^7^
After the removal of outliers for threat mean amplitude, there was a marginal negative association between neutral mean amplitude and GAD symptoms and a positive association between neutral LPP slope and GAD symptoms. After removal of threat LPP slope outliers, there was a marginal positive association between the neutral LPP slope and GAD symptoms. The removal of neutral mean amplitude outliers led to a positive association between the neutral LPP slope and GAD symptoms. Across all models, the positive association between threat mean amplitude and GAD symptoms and the negative association with the threat LPP slope and GAD symptoms remained consistent. As such, we chose to report the models that included all data.

^8^
The removal of outliers from threat and neutral mean amplitude, and threat LPP slope led to a positive association between neutral LPP slope and GAD symptoms. The removal of outliers from neutral LPP slope led to a marginal positive association between neutral LPP slope and GAD symptoms. Further, the removal of mean threat amplitude outliers led to a marginal negative association between neutral mean amplitude and GAD symptoms. Together, the removal of outliers from our models tended to strengthen the association between threat LPP slope and threat mean amplitude and inconsistently strengthened the marginal positive association between the neutral LPP slope and GAD symptoms. As such, we chose to report the data without outliers removed.

^9^
While emotional dysregulation and anxiety are distinct constructs, emotional dysregulation is one mechanism that is proposed to drive perpetuate GAD symptoms (Mennin et al. 2005). Monopoli & colleagues found those who reported heightened emotion intensity had a larger LPP intercept (measured at 500 ms) and smaller LPP slope from 500–2000 ms post‐stimulus presentation. Notably, this pattern of LPP responses closely aligns with the current study, which finds that more positive threat slopes during the early LPP, but more negative threat slopes during the later LPP, are each associated with GAD symptoms. Although this pattern of findings is similar, there are several methodological differences between the current study and Monopoli and colleagues. Notably, the current study examined the early LPP (400–700 ms), where Monopoli and colleagues examined the LPP in later windows. Specifically, Monopoli and colleagues computed mean amplitudes for 250 ms windows ranging from 500–2000 ms post stimulus onset. As such, the earliest slope is measured from the mean of 500–750 ms to 750–1000 ms, or after the peak of the LPP for low‐anxiety individuals in our study.
