# Supplementary material for: The Temporal Dynamics of Attention to Threat and GAD Symptoms: A Study of LPP Slopes
Source: Psychophysiology. 2025 Sep 30;62(10):e70157. doi: 10.1111/psyp.70157 (PMC12481178; doi:10.1111/psyp.70157)
Supplement: Supplementary file 1 — Table S1: Model fits for multilevel models. Table S2: Stepwise regression results. Table S3: Model results predicting GAD‐7 Sx across overlapping windows. [file PSYP-62-e70157-s001.docx]

**S1. Model Building**

To build the multi-level models, a stepwise procedure was followed. First, we estimated an intercept only model to determine an intraclass coefficient (*ICC*). The *ICC* enabled the calculation of the proportion of variance in LPP amplitude that was due to between subject variability (i.e., individual differences) (Volpert-Esmond et al., 2021) (model 1 in Table S1.

LPP Amplitude ∼ 1 + (1 | Participant)

We then estimated a model where time was entered as both a fixed and random effect (model 2 in Table S1).

LPP Amplitude ∼ Time + (1 + Time | Participant)

In a subsequent model, we also added image valence as a fixed effect in addition to time. The slope of time was still allowed to vary across participants as a random effect (model 3 in table S1).

LPP Amplitude ∼ Time + Image Valence + (1 + Time | Participant)

Image valence was then added as a random effect (model 4 in Table S1).

LPP Amplitude ∼ Time + Image Valence + (1 + Time + Image Valence | Participant)

Then, time and image valence were allowed to interact as a fixed effect while time and image valence were allowed to vary across participants (model 5 in Table S1).

LPP Amplitude ∼ Time + Image Valence + Time × Image Valence +

(1 + Time + Image Valence | Participant)

The final model consisted of predictors for time (e.g., 450 ms), image valence (threat, neutral), and their interaction. Time, image valence, and their interaction were also entered as random effects (model 6 in Table S1).

LPP Amplitude ∼ Time + Image Valence + Time × Bin +

(1 + Time + Image Valence + Time × Image Valence | Participant)

**Table S1. Model fits for Multi-Level Models**

|  | **BIC** | **Log Likelihood** |
| --- | --- | --- |
| **400-700 ms** |  |  |
| Model 1 | 336577.7 | -168272.2 |
| Model 2 | 324170.3 | -162051.9 |
| Model 3 | 294685 | -147303.7 |
| Model 4 | 245185.9 | -122537.6 |
| Model 5 | 245123.9 | -122501 |
| Model 6 | **237960.4** | -118897.1 |
|  |  |  |
| **700-2000 ms** |  |  |
| Model 1 | 1380902 | -690432.4 |
| Model 2 | 1339420 | -669672.6 |
| Model 3 | 1294255 | -647083.7 |
| Model 4 | - | - |
| Model 5 | 1116318 | -558090.3 |
| Model 6 | **1079257** | -539534.6 |

*Note.*

Model 1 = LPP Amplitude ∼ 1 + (1 | Participant),

Model 2 = LPP Amplitude ∼ Time + (1 + Time | Participant),

Model 3 = LPP Amplitude ∼ Time + Image Valence + (1 + Time | Participant)

Model 4 = LPP Amplitude ∼ Time + Image Valence + (1 + Time + Image Valence | Participant)

Model 5 = LPP Amplitude ∼ Time + Image Valence + Time × Image Valence + (1 + Time + Image Valence | Participant)

Model 6 = LPP Amplitude ∼ Time + Image Valence + Time × Bin + (1 + Time + Image Valence + Time X Image Valence | Participant)

**S2. Alternate Time Windows**

We also assessed the LPP from 200 – 700 ms. From 200 to 700 ms, between-subjects variability (i.e., individual differences) accounted for approximately 58% of the variance in LPP amplitude (*ICC* = .58). In this model, there was a fixed effect of Time *t*(107412) = 8.11, *p* < .001, such that amplitudes became more positive over time from 200 – 700 ms. There was also a main effect of Image Valence, such that amplitudes were greater for threat (*M* = 2.69, *SE* = 0.46) than neutral images (*M* = 0.01, *SE* = 0.56), *t*(107412) = 2.52, *p* = .012. There was also a significant interaction of Time × Image Valence, such that the rate of change in amplitudes, on average and across all participants, were more positive for threat than neutral pictures during this time window, *t*(107412) = 8.83, *p* < .001. The LPP slope to threat images was related to GAD symptoms after controlling for mean amplitude *t*(83) = 2.01, *p* = .046^[[1]](#footnote-1)^. However, the LPP slope to threat images was not related to GAD symptoms in subsequent models that controlled for mean amplitude and slope to neutral images as well as gender. Results from stepwise regressions with anxiety scores are shown in Table S2.

**Table S2.**

*Stepwise Regression Results*

|  | 200 – 700 ms | | | |  |
| --- | --- | --- | --- | --- | --- |
| Predictor | Model 1 | Model 2 | Model 3 | Model 4 |  |
| Threat mean amplitude | **.26*** | **.24*** | **.45*** | .30 |  |
| Threat Slope |  | **.22*** | .22 | .21 |  |
| Neutral mean amplitude |  |  | -.26 | -.12 |  |
| Neutral Slope |  |  | -.05 | -.14 |  |
| Gender |  |  |  | **.24**** |  |
| *R*^2^ | .05 | .09 | .11 | .19 |  |
| Δ*R*^2^ | .05 | **.04*** | .02 | .**08**** |  |

Note. Standardized regression coefficients (βs) and R2 are reported. p values < .05 are shown in bold. ****p* < .001; ***p* < .01; **p* < .05; †*p* < .10 (two-tailed). Gender was dummy coded such that male = 0 and female = 1.

**S3. Overlapping Windows**

To better understand the time course of the LPP response, overlapping 100 ms windows from 400 – 2000 ms (e.g., 400 – 500 ms, 450 – 550 ms) were examined. Specifically, the same multilevel model that included the interaction between time and image valence as both fixed and random effects at each time interval was fit. LPP slope estimates and mean amplitudes were again extracted.

To estimate the precise timing of any association between GAD symptoms and the slope, multilevel models were conducted on overlapping 100 ms window durations throughout the LPP response. At the earliest estimated window (400 – 500 ms), there was a significant Time × Image Valence interaction, *t*(21522) = 3.78, *p* < .001, such that the threat LPP slope was larger than neutral slope. In the 600 – 700 ms window model, we again observed a significant Time × Image Valence interaction, except that this interaction effect was in the opposite direction as was seen in the 400 ms model, such that threat slope was significantly more negative than the neutral slope across all participants from 600 – 700 ms, *t*(21312) = -2.30, *p* = .021. Finally, in the 1850 – 1950 ms model, we again observed a significant Time × Image Valence interaction, *t*(21312) = 2.45, *p* = .014, such that threat slope was more positive than the neutral image slope.

For each interval estimated, GAD symptoms were modeled as being predicted by gender and the four LPP variables we have been examining (i.e., threat slope, threat mean amplitude, neutral slope, and neutral mean amplitude). In this set of analyses, threat slope was positively related to anxiety symptoms from 500 – 600 ms, β = .25, *t*(80) = 2.37, *p* = .020, as well as from 1550 – 1650 ms, β = -.22, *t*(80) = -2.12, *p* = .037. Neutral slope was negatively related to GAD symptoms from 1200 – 1300 ms, β = -.20, *t*(80) = -2.01, *p* = .047. Threat mean amplitude was related to anxiety in each 100 ms window with onsets ranging from 500 ms to 1100 ms, as well as in the 1300 – 1400 ms window. Importantly, none of the associations with either the LPP slope to threat images survive a Bonferroni correction for multiple comparisons. The results from the overlapping intervals are shown in Table S3.

**Table S3**

*Model Results Predicting GAD-7 Sx Across Overlapping Windows*

| Window (ms) | Threat Slope | Threat Mean Amplitude | Neutral Slope | Neutral Mean Amplitude | Gender | Total *R*^2^ |
| --- | --- | --- | --- | --- | --- | --- |
| 400 – 500 | .07 | .24 | .00 | -.15 | **.31**** | **.15**** |
| 450 – 550 | .11 | .29† | .07 | -.16 | **.28*** | **.18**** |
| 500 – 600 | **.25*** | **.37*** | .01 | -.16 | **.26*** | **.24**** |
| 550 – 650 | .08 | **.41*** | .05 | -.16 | **.29**** | **.21**** |
| 600 – 700 | .08 | **.44**** | <.01 | -.19 | **.27*** | **.23***** |
| 700 – 800 | **.**07 | **.42**** | -.03 | -.21 | **.29**** | **.24***** |
| 750 – 850 | .06 | **.49***** | .05 | -.22† | **.28**** | **.26***** |
| 800 – 900 | -.01 | **.46***** | .04 | -.21 | **.30**** | **.26***** |
| 850 – 950 | -.09 | **.40**** | -.06 | -.21† | **.32**** | **.26***** |
| 900 – 1000 | -.18 † | **.41***** | -.01 | -.20† | **.30**** | **.27***** |
| 950 – 1050 | -.09 | **.36**** | .03 | -.15 | **.31**** | **.23***** |
| 1000 – 1100 | -.03 | **.32*** | .17† | -.12 | **.31**** | **.24***** |
| 1050 – 1150 | -.08 | **.30*** | .04 | -.06 | **.30**** | **.21***** |
| 1100 – 1200 | -.05 | **.31*** | .06 | -.03 | **.30**** | **.21**** |
| 1150 – 1250 | - | - | - | - | - | - |
| 1200 – 1300 | .06 | .22 | **-.20*** | <.01 | **.32**** | **.22**** |
| 1250 – 1350 | -.13 | .24† | .13 | -.02 | **.33**** | **.20**** |
| 1300 – 1400 | .17 | **.27*** | -.06 | -.01 | **.31**** | **.20**** |
| 1350 – 1450 | -.04 | .23† | .15 | .01 | **.31**** | **.20**** |
| 1400 – 1500 | -.02 | .19 | .03 | .05 | **.32**** | **.18**** |
| 1450 – 1500 | .13 | .18 | -.02 | .05 | **.31**** | **.19**** |
| 1500 – 1600 | - | - | - | - | - | - |
| 1550 – 1650 | **-.22*** | .16 | -.06 | .02 | **.33**** | **.23***** |
| 1600 – 1700 | - | - | - | - | - | - |
| 1650 – 1750 | -.15 | .11 | .04 | .11 | **.31**** | **.19**** |
| 1700 – 1800 | .02 | .11 | -.06 | .09 | **.34**** | **.16*** |
| 1750 – 1850 | .14 | .15 | .01 | .07 | **.33**** | **.19**** |
| 1800 – 1900 | -.13 | .18 | -.01 | .05 | **.34**** | **.18**** |
| 1850 – 1950 | .04 | .13 | -.08 | .07 | **.33**** | **.17**** |
| 1900 – 2000 | - | - | - | - | - | - |

*Note*. Each model included all five predictors (i.e., the variables named within the columns) concurrently. Standardized regression coefficients are reported for slopes and mean amplitudes. Bolded values indicate *p* < .05. † = *p* < .10, * = *p* <.05, ** = *p* < .01, *** *p* < .001. Results from models that failed to converge are indicated via *-* across all cells.

**S4. Overlapping Windows Discussion**

To further understand associations with the LPP response, LPP slopes and amplitudes were estimated across overlapping 100 ms windows. Across all participants, threat slope was more positive than neutral slope from 400 – 500 ms. The overall effect suggests that normative processing of threat images is distinguished from that of neutral images by a faster increase in the LPP from 400 – 500 ms. Further supporting this interpretation, neither LPP slope nor mean amplitude predicted GAD symptoms in this window—such an early response stage may be more or less similar across individuals and nondiagnostic. However, such a normative response may start to give way to individual differences only around 100 ms later: in the 500 – 600 ms window, where a greater threat LPP slope was positively related to GAD symptoms. Additionally, LPP mean amplitude to threat was positively associated with GAD symptoms continuously across windows from 500 – 1200 ms. These results suggest that early in the LPP response (400 – 500 ms), there is a normative increase of the LPP in response to threat. However, at approximately 500 ms, there is a critical point in the attentional processing that differentiates those reporting greater levels of GAD symptoms. At this point, individuals experiencing more anxiety tend to continue deploying attention to threat, as reflected by larger threat LPP slope. Conversely, individuals experiencing less anxiety have less of a threat LPP slope at this time. Given that mean amplitude, but not slope, was related to GAD symptoms in the overlapping time windows following 500 ms, it is possible that the rapid change in the LPP to threat (LPP slope) from 500 – 600 ms drives the later association seen with mean amplitude and GAD symptoms in later measurement windows. Together, the LPP from 400 – 700 ms is characterized by a normative deployment of attention to threat at the early portion of the window (400 – 500 ms). However, LPP slopes that remain more positive after 500 ms are associated with GAD symptoms. Although the overlapping window analysis was co-registered; these analyses consisted of multiple comparisons, and no associations between the LPP slope and GAD symptoms survived correction for these multiple comparisons. Nonetheless, it is our hope this approach provided an initial step to distinguishing when the time course of normative processing of threat begins to differ from the time course of threat processing in those who report higher GAD symptoms, and to lay the groundwork for future work on this topic.

**References**

Volpert-Esmond, H. I., Page-Gould, E., & Bartholow, B. D. (2021). Using multilevel models for the analysis of event-related potentials. *International Journal of Psychophysiology*, *162*, 145–156. https://doi.org/10.1016/j.ijpsycho.2021.02.006

1. The removal of threat mean amplitude outliers led to a non-significant association between threat mean amplitude and GAD symptoms. The relationship between the threat LPP slope and GAD symptoms was unaffected and they remained positively associated. [↑](#footnote-ref-1)
